# 1157. Immune Persistence and Booster Response of a Quadrivalent Meningococcal Conjugate Vaccine (MenACYW-TT) 5 Years After Primary Vaccination of Adults ≥59 Years of Age

**DOI:** 10.1093/ofid/ofad500.997

**Published:** 2023-11-27

**Authors:** Corwin A Robertson, Katherine Galarza, Jeffry Jacqmein, Alexandre Selmani, Philipp Oster

**Affiliations:** Sanofi Pasteur, Swiftwater, Pennsylvania; Sanofi , Swiftwater, Pennsylvania; University of Florida Health Family Medicine, Jacksonville, Florida; Sanofi, Swiftwater, Pennsylvania; Sanofi Pasteur, Swiftwater, Pennsylvania

## Abstract

**Background:**

MenACYW-TT (MenQuadfi®, Sanofi) is a quadrivalent (serogroups A, C, W, and Y) meningococcal tetanus toxoid conjugate vaccine. It is approved for use in persons aged ≥2 years in the US and persons aged ≥1 year in Europe and certain other countries; trials in infants as young as 6 weeks are ongoing. The second stage of a 2-stage study of MenACYW-TT, reported herein, evaluated immune persistence among older adults who received primary vaccination with either quadrivalent meningococcal polysaccharide vaccine (MSPV4) or MenACYW-TT five years earlier at age ≥ 56 years. The immunogenicity and safety of a MenACYW-TT booster dose given to this population were also evaluated.

**Figure:**
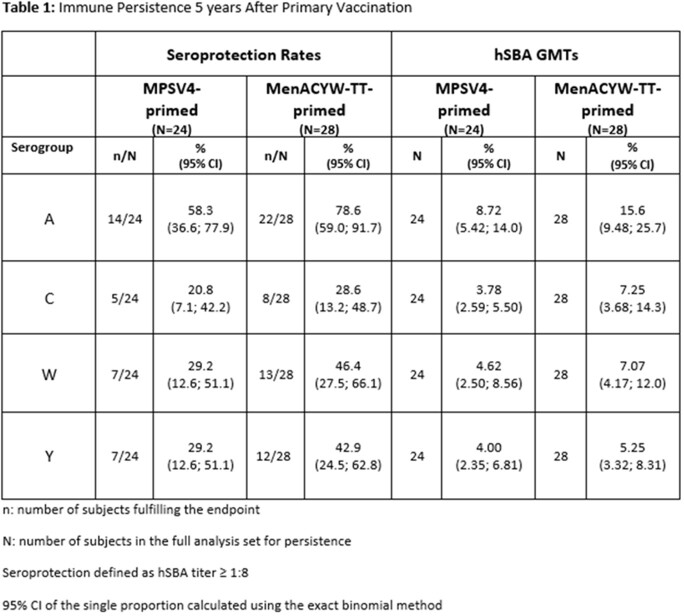

**Figure:**
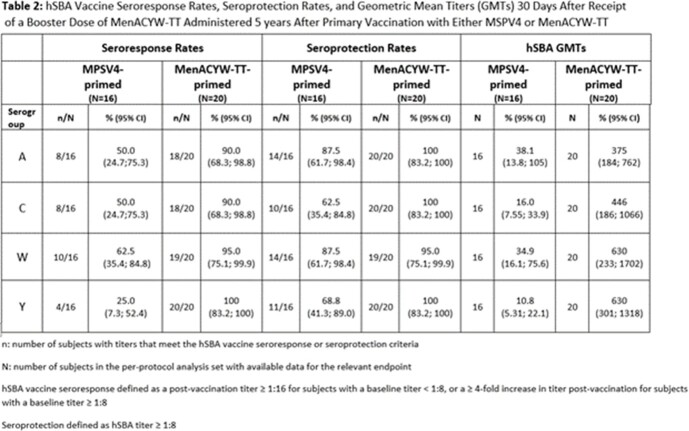

**Methods:**

This was a Phase 3, 2-stage, randomized, open-label, multi-center study (NCT04142242) of adults aged ≥59 years who participated in previous studies of MenACYW-TT vs MPSV4 (NCT01732627 and NCT02842866). The study was conducted in the US and Puerto Rico. Immune persistence and response were assessed with a serum bactericidal assay using human complement (hSBA). Safety data were collected for up to 30 days after booster vaccination. All analyses were descriptive.

**Results:**

Of the initial study population (N=471), 70 adults participated in Stage II of the study. At 5 years post-primary vaccination, seroprotection (hSBA ≥ 1:8) rates (SPRs) and hSBA geometric mean titers (GMTs) declined in both MenACYW-TT- and MPSV4-primed subjects, with SPRs and hSBA GMTs for serogroups A, C, W, and Y trending higher in MenACYW-TT- vs MPSV4-primed subjects (Table 1). Thirty days after booster vaccination, hSBA GMTs and SPRs increased from pre-booster for all serogroups and were, along with seroresponse rates, higher in MenACYW-TT-primed subjects compared to MPSV4-primed subjects (Table 2). Rates of AEs following booster vaccination were similar regardless of priming vaccine. No safety concerns were identified.

**Conclusion:**

Five years after primary vaccination, immune persistence tended to be greater for MenACYW-TT vs MPSV4. A MenACYW-TT booster was well tolerated and immunogenic in adults ≥ 61 years, when administered 5 years after primary vaccination with either MPSV4 or MenACYW-TT.

**Disclosures:**

**Corwin A. Robertson, MD, MPH, FACP**, Sanofi: Sanofi employee|Sanofi: Sanofi's Employee|Sanofi: Stocks/Bonds **Katherine Galarza, MD**, Sanofi: Sanofi employee|Sanofi: Stocks/Bonds **Alexandre Selmani, PhD**, Sanofi: Sanofi employee|Sanofi: Stocks/Bonds **Philipp Oster, MD**, Sanofi: Sanofi employee

